# Calibration of Thermal Viscoelastic Material Models for the Dynamic Responses of PVB and SG Interlayer Materials

**DOI:** 10.3390/polym16131870

**Published:** 2024-06-30

**Authors:** Jon Knight, Hani Salim, Hesham Elemam, Ahmed Elbelbisi

**Affiliations:** Civil and Environmental Engineering, University of Missouri, Columbia, MO 65211, USA; jtktk5@mail.missouri.edu (J.K.); elemamh@missouri.edu (H.E.)

**Keywords:** viscoelastic, material model, laminated glass interlayer, polyvinyl butyral, SentryGlas^®^ polymer, finite element modeling

## Abstract

Laminated glass interlayer materials polyvinyl butyral (PVB) and SentryGlas^®^ (SG, kuraray, Houstan, TX, USA) exhibit thermal viscoelastic behavior under dynamic tensile loading. Significant temperature and strain rate effects on the behavior of these interlayer materials pose a challenge for accurately modeling the dynamic response of laminated glass. Many researchers have simplified their approaches by modeling the response of the interlayer material using a bilinear approximation or established hyperelastic models. However, temperature and strain rate effects can be captured using the three-network viscoplastic (TNV) model. Therefore, the objective of this study is to calibrate material models for the thermal viscoelastic dynamic responses of PVB and SG interlayer materials. Uniaxial tensile tests were performed at strain rates of 2, 20, and 45 s^−1^ and temperatures of 0, 23, and 60 °C, and material models were calibrated using the experimental data. Finite element analysis using the calibrated material models successfully predicted the dynamic responses of PVB and SG under the experimental test conditions within a 10% error margin. This suggests that the calibrated models using the TNV model represent significant improvements over existing approaches to modeling the dynamic response of laminated glass. Similar procedures can be applied to other thermoplastics, laying the groundwork for establishing a standard calibration guide.

## 1. Introduction

The surge in terrorist attacks targeting densely populated urban areas in recent years has heightened concerns about building safety, underscoring the critical importance of designing building envelopes, which increasingly incorporate large glass curtain wall systems. In the event of failure in such systems, sharp glass fragments may become projectiles, posing a serious threat to occupants and potentially causing injuries or fatalities [1]. Studies indicate that the majority of nonfatal injuries sustained during bomb blasts stem from flying fragments originating from architectural glazing [2,3]. Laminated glass (LG) has been found to be effective at mitigating these risks by retaining glass fragments on a polymeric interlayer upon fracture [4,5,6]. Additionally, after glass cracking, the ductile polymer interlayer deforms significantly as a continuous membrane providing post-cracking energy absorption [7]. The shear behavior of the interlayer material affects the deformability and load-bearing capacity of laminated glass plates [8], as well as their post-breakage behavior [9,10,11]. In other words, the polymeric interlayer governs the general behavior of laminated glass, highlighting its importance for the adequate design and calculation of laminated glass structural elements. Interlayer materials are viscoelastic, meaning their stiffness and mechanical response depend on load duration and working temperature [12,13,14]. Therefore, the mechanical properties of laminated glass also depend on at least these two factors [8,15,16].

Larcher et al. [17] state that the failure process of a laminated glass sheet can be divided into five phases: (1) elastic behavior of glass plies, (2) outer glass ply cracks while the interlayer is not damaged, (3) inner glass ply fails and the interlayer behaves elastically, (4) interlayer deforms plastically and splinters are kept together by the interlayer, and (5) the interlayer fails at ultimate strength or is cut by the glass shards. Extensive research has been conducted on the elastic behavior of laminated glass, making modeling phase (1) with numerical models straightforward. However, phases (3) through (5), largely dominated by the mechanical properties of the viscoelastic polymer interlayer, pose greater challenges. Thus, calibrating material models to accurately predict the dynamic responses of polymeric interlayer materials is critical for the analysis and design of laminated safety glass.

Kott and Vogel define the failure modes of laminated glass panels to specify their remaining structural capacity [18]. These preconditions, along with four-point bending tests and previous impact tests, form the foundation for developing a new mechanical model. Various geometric parameters are evaluated for panes supported on two sides, considering both limit states: load-carrying capacity (LCC) and residual strength capacity (RSC). The types of fractures and the corresponding yield line mechanisms are also analyzed. This comprehensive approach enables the improvement of RSC and provides an analytical verification.

Analysis of the dynamic response of laminated glass windows through full-scale dynamic testing is both extremely costly and time-intensive. Finite element modeling (FEM) presents itself as an efficient and cost-effective tool for such analysis. However, modeling laminated glass poses challenges due to the significantly divergent mechanical responses of its constituent materials. Glass is characterized by its brittleness, high modulus of elasticity, and low ductility. Its response under tensile stress is generally linear elastic until sudden brittle fracture. In contrast, interlayer materials are viscoelastic polymers with a relatively low modulus of elasticity and high ductility. The response of interlayer materials under tensile stress involves highly nonlinear mechanical behavior with significant strain rate and temperature dependencies [1,6,15,17,19,20,21,22,23,24,25,26,27,28,29,30,31,32,33,34,35,36,37,38].

In the literature, various approaches have been explored for modeling laminated glass windows due to their inherent complexity as composite materials composed of constituent materials with vastly different mechanical properties [6,39]. Among the finite element models utilized for laminated glass, researchers often combine shell elements with membrane elements [17,40,41,42,43]. Glass is typically modeled using shell elements as linear elastic materials with failure at a critical strain limit [17,40,41,42,43] or Rankine stress limit [42], or with piecewise linear plasticity models to allow small amounts of plastic strain [40,43]. However, modeling of the interlayer material varies.

Some researchers have approximated the polyvinyl butyral (PVB) interlayer as linear elastic, neglecting large nonlinear strain behavior and viscoelasticity [43]. Others have employed widely used hyperelastic laws available in LS-DYNA, such as the Blatz-Ko, Mooney–Rivlin, and Ogden materials, to model the PVB interlayer [40,41,44]. While hyperelastic material models capture the nonlinear elastic behavior of materials based on their stress–strain responses, they do not inherently account for strain rate or temperature dependencies and do not accurately capture the stiff initial linear elastic region of polymers under high strain rates.

**Figure 1:**
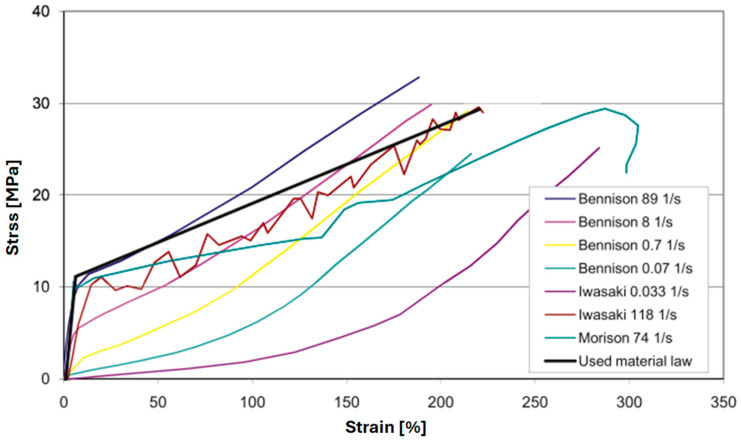
Elastic-plastic material law used by Larcher et al. [8] compared with experimental test data of PVB at different strain rates (Morrison [36], Iwasaki [50], Bennison [22]).

Larcher et al. [17] noted that the behavior of PVB under higher strain rates is more elastic with hardening than viscoelastic, and accordingly modeled PVB interlayer with a bilinear elastic–plastic material law, as shown in Figure 1. Lusk et al. [45] modeled laminated glass windows under blast loading using the LS-DYNA material model 32 “MAT-LAMINATED_GLASS”, which treats the response of the interlayer as bilinear with input data of Young’s modulus, Poisson’s ratio, yield stress, and plastic hardening modulus. Many other researchers have employed this elastic–plastic bilinear approach to modeling the response of laminated glass interlayer materials [37,46,47,48,49]. However, while the elasto-plastic material model approach approximates the stiff initial linear region at high strain rates, it does not account for the significant strain rate and temperature dependencies in the mechanical responses of interlayer materials. Furthermore, it does not accurately capture more complex material responses, such as that of the interlayer material SentryGlas^®^ (SG).

Analytical approaches have been made to develop master curves for the mechanical characterization of viscoelastic materials that incorporate time and temperature variables. Hána et al. formulated master curves for two types of interlayers, such as PVB-based TROSIFOL^®^ BG R20 and EVA-based EVALAM^®^, using the results of static and dynamic single-lap shear tests to identify parameters for a generalized Maxwell chain model with an incorporated temperature shift factor to describe their time- and temperature-dependent relaxation functions [51]. Similarly, Centelles et al. performed relaxation tests on seven polymeric films used as laminated glass interlayer materials and developed master relaxation curves using a generalized Maxwell model and the time–temperature superposition principle [52]. Liu et al. [29] performed tensile tests on PVB material under a range of strain rates and proposed sets of constitutive equations using the ZWT model. The parameters of the constitutive equations were fitted to each individual strain rate and tested without considering temperature effects. Chen et al. performed uniaxial tensile tests on the ionomer interlayer (SG) at a wide range of strain rates and temperatures and proposed dynamic constitutive equations for different temperatures based on a modified G’Sell model [24]. The results of these analytical approaches did not yield material models readily available for implementation into finite element analysis and were not validated by numerical simulations.

The mechanical responses of thermoplastics, such as PVB and SG, are typically well captured with a three-network frame [53]. Two efficient candidates are the three-network (TN) model and the three-network viscoplastic (TNV) model [54]. Both the TN and TNV models are powerful models capable of capturing strain rate and temperature effects. The three-network (TN) model is specifically developed for thermoplastic materials, and as such, the individual networks implemented are immutable. Shahin et al. [55] calibrated the TN model to successfully model the relaxation response of high-density polyethylene (HDPE) flanges with an error less than 10%. Similar model calibrations for HDPE that are solely suitable for constant loads, e.g., creep relaxation, were proposed by Lai et al. [56] and Elluech et al. [35,57]. The three-network viscoplastic (TNV) model is more versatile and is capable of capturing the time dependence, the pressure dependence of plastic flow, the pressure-dependent bulk modulus, the volumetric plastic flow, and anisotropy. The individual networks making up the TNV model can be selected and optimized to fit a wide range of material behaviors. Recently, Almomani et al. [53] calibrated the TNV model to predict the thermal–viscoelastic behaviors of HDPE under monotonic and cyclic loads. The model was implemented into FEA analysis and was successfully able to predict the HDPE behavior under static, quasi-static, and dynamic loads and at temperatures from 23 °C to 95 °C.

Based on the aforementioned studies, it can be concluded that the TNV model shows promise for capturing the versatile, nonlinear strain rate and temperature-dependent behaviors of laminated glass interlayers. However, to date, no studies have been found that investigate its calibration on PVB, SG, or any laminated glass interlayer materials for the monotonic tensile response at various strain rates and temperatures. Therefore, the objective of this study is to calibrate the TNV model to capture the thermo-viscoelastic dynamic responses of PVB and SG interlayer materials under a wide range of strain rates and temperatures. The calibrated material models will then be validated by finite element simulations.

## 2. Experimental Program

In this section, the interlayer materials tested are described in detail. Test specimen preparations are provided. An overview of two tensile test methodologies, pneumatic testing and drop-weight testing, is presented. Data acquisition and processing are discussed. Finally, the experimental test matrix is detailed.

### 2.1. Material Description

Polyvinyl butyral (PVB), a solid thermoplastic resin invented in 1927, was the first material used as an interlayer for laminated glass, and it remains widely used today in various structural applications. PVB is produced through the reaction of polyvinyl alcohol with butyraldehyde. This random amorphous polymer consists of three monomers, each contributing specific properties: hydrophobic and elastic vinyl butyral (~80 wt%) provides processability, while hydrophilic vinyl alcohol (~18 wt%) and vinyl acetate (~2 wt%) ensure high adhesion to inorganic materials such as glass [58].

Several experiments have been conducted to understand the dynamic response of PVB. Nawar et al. showed that the mechanical response of PVB is highly strain-rate-dependent and that the quasi-static behavior resembles a hyperelastic material such as rubber, and at high strain rates, the behavior transitions to a viscoelastic bilinear response [37]. Andreozzi et al. performed experiments with a rheometer and found that a PVB between 0 °C and 50 °C is in the glass transition region. Therefore, its dynamic modulus is highly temperature-dependent in the typical in situ temperature ranges. At high temperatures, the dynamic modulus is extremely low, and the behavior is hyperelastic. At low temperatures, PVB behaves like an elasto-plastic material and exhibits some brittleness [26].

SentryGlas^®^ (SG), sometimes referred to as an “ionoplast interlayer”, is the only commercially available ionomer-based interlayer material for laminated glass. Developed by DuPont for use in structural safety glazing, SG offers much greater rigidity, higher strength, and better ductility compared to PVB [24]. Since its market release in 1998, structural engineers have commonly used SG as a replacement for PVB to improve the mechanical response of laminated glass.

An ionomer is a polymer that typically has an ionic content of no more than 10 mole percent. The general chemical structure of an ionomer consists of a hydrocarbon containing neutralized pendant acid groups [59]. SG is formed through a process in which a copolymerization of ethylene with methacrylate is cured with metal ions [60]. Due to its semi-crystalline chemical structure and crosslinking, SG offers superior mechanical properties compared to traditional amorphous polymer interlayers. Furthermore, SG provides excellent adhesion to glass and metals because of its ionic interactions and chemical bonding.

Several studies have been performed to investigate the thermo-viscoelastic properties of SG interlayer materials. Zhang et al. [23] performed uniaxial tensile tests on SentryGlas Plus (SGP) interlayer material under a wide range of strain rates and found that SGP exhibited an elasto-plastic behavior at all strain rates, ranging from quasi-static rates to extremely high dynamic rates. Due to its semicrystalline structure, SG undergoes a yielding event after a stiff initial linear region and subsequent strain softening as the polymer chains begin to slide past each other within the amorphous regions and the crystalline domains continue to deform elastically. The strain rate effect predominantly affects the initial response, resulting in increased yield stress with increasing strain rate. The tests also found that SGP material became less ductile as the strain rate increased. Chen et al. [24] performed uniaxial tensile tests on SG interlayer material at a wide range of strain rates and temperatures and found that the initial modulus decreased with the increase in temperature; the yield stress decreased sharply with the increase in temperature, especially at high strain rates; and the yield strain showed limited dependence on the strain rate. The glass transition temperature of SG was approximately 50–55 °C [25].

Laminated glass manufacturers typically receive interlayer materials in sheets, with specific thicknesses of rolls. The sheets are then cut to size and placed in between the glass plies, and the composite window is processed in an autoclave under high pressures and temperatures. The curing process has been shown to have significant effects on the material responses of laminated glass interlayers. Accordingly, PVB and SG sheets that had already gone through the curing process were acquired.

### 2.2. Specimen Preparations

**Figure 2:**
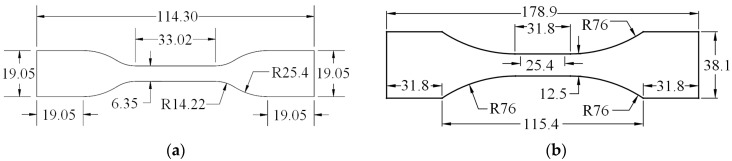
Specimen preparation: (**a**) quasi-static specimen geometry and (**b**) dynamic specimen geometry (dimensions in mm). Reproduced from [38] Polymers, 2024.

In this work, the sample geometry for the intermediate strain rate testing at a 2 s^−1^ strain rate, performed utilizing the pneumatic testing machine, was the ASTM D638-14 [61] standard Type IV specimen, as shown in Figure 2a. A modified ASTM D638-14 standard Type I geometry was employed for the high-strain-rate testing at 20 s^−1^ and 45 s^−1^ strain rates, which was performed utilizing a drop-weight testing machine. The end area was increased, as shown in Figure 2b, and aluminum tabs were adhered to the end tabs of the specimens to prevent tearing-out failure of the specimen under impact loading. Steel cutting dies were fabricated for both specimen geometries to ensure precise dimensions [38]. For each specimen, the 1 inch central gauge length, L_g_, was marked with thin black lines/points using a permanent marker pen prior to testing to enable the strain to be monitored during the test using a high-speed camera. Steel digital calipers were used to measure the thickness and width of the test section at three locations, to an accuracy of 0.0005 in.

### 2.3. Test Setups

**Figure 3:**
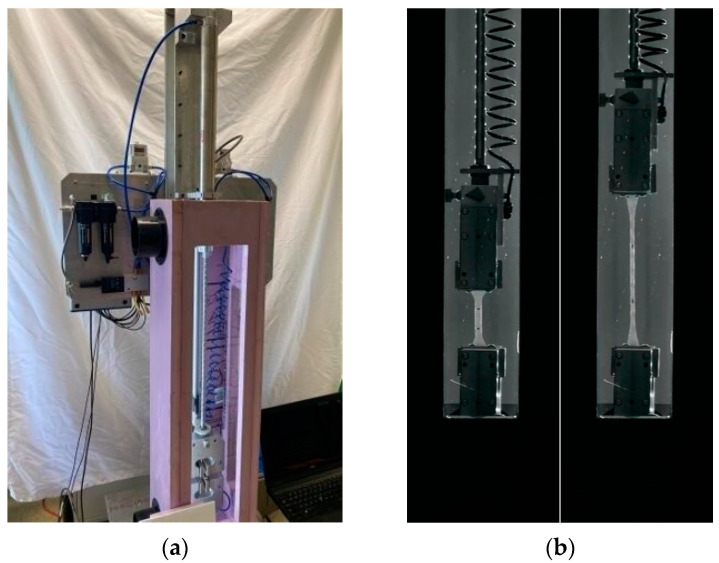
Pneumatic test setup: (**a**) pneumatic testing machine with temperature enclosure, and (**b**) select frames from video of an intermediate strain rate test on PVB: start of testing (**left**) and mid-test (**right**).

For intermediate strain rate testing, a pneumatic testing machine was designed and fabricated to be able to perform temperature-controlled tensile tests at strain rates ranging from quasi-static rates to 5 s^−1^, as shown in Figure 3a. The device was equipped with a load cell with a capacity of 250 pounds (1112 N) and was operated under displacement control by a LabView program. The elongation of the specimens throughout testing was tracked using an edgertronic SC1 model high-speed camera, capturing frames at a rate of 3000 frames per second. Select frames from a video of an intermediate strain rate test on PVB are shown in Figure 3b. A temperature enclosure was built around the testing frame to enable us to perform temperature-controlled testing.

**Figure 4:**
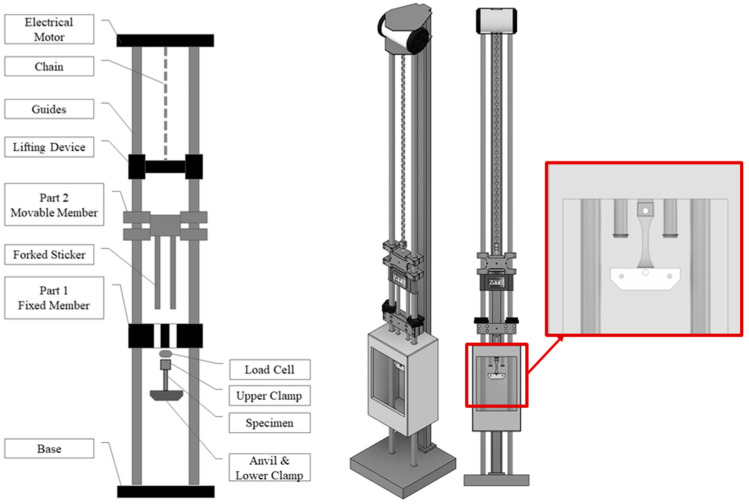
Drop weight test set-up, reproduced from [38] Polymers, 2024.

The high-strain-rate tensile testing in this research was performed using a modified drop-weight apparatus; see Figure 4. This machine was originally invented for impact testing of robust materials like metals or composites. For the performance of high-strain-rate examinations on polymers, multiple novel components were designed, fabricated, and implemented. An illustrative diagram of the machine, with the critical parts identified, is presented in Figure 4. A moveable member with variable weight, shown in Figure 4, was equipped with forked strikers, as shown in Figure 4, and installed onto the guide rails. A fixed member with bore holes was fabricated to allow the strikers to pass through and impact the lower clamp, which was attached to the bottom tab of the test specimen, as well as to stop the moveable member after the specimen had failed. The upper tab of the specimen was attached to a load cell with the upper clamp. A piezoelectric load cell (Omegadyne model LC213-500; Omegadyne, Nowalk, CT, USA; Edgertronic, Campbell, CA, USA) was used to record the load at a rate of 3000 data points per second. The elongation of the specimens throughout testing was tracked using an edgertronic SC1 model high-speed camera, capturing frames at a rate of 3000 frames per second. The load from the piezoelectric load-cell was recorded using a National Instruments USB-6351 data acquisition system.

**Figure 5:**
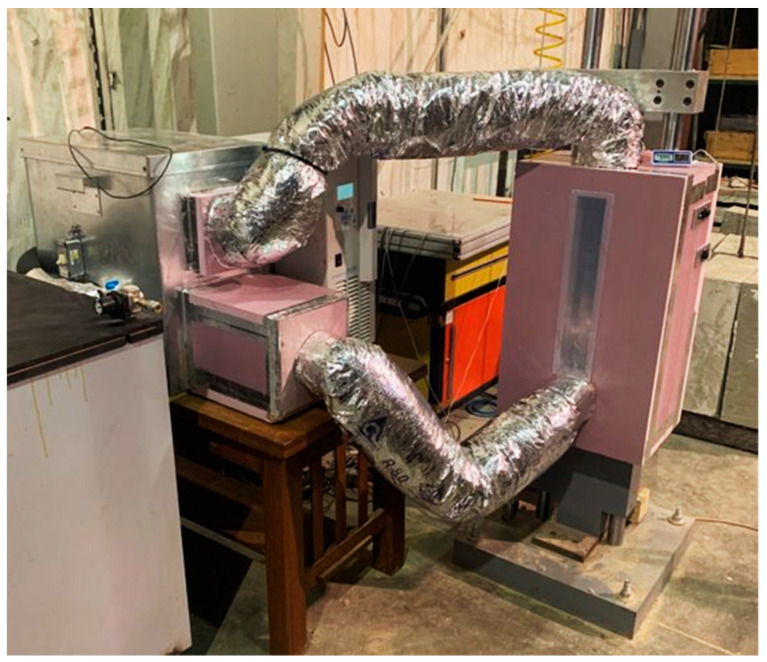
Temperature control system with drop weight testing machine.

A temperature enclosure was built around the testing frame to be able to perform temperature-controlled testing. The temperature control system utilized was comprised of a Thermo Fisher Scientific™ Accel 500 LT cooling/heating chiller with a working temperature range of −25 °C to +80 °C with an accuracy of ±0.5 °C, an industrial-grade air ventilator, and insulated ducts. The temperature control system with the drop weight testing machine is shown in Figure 5.

### 2.4. Data Processing

The load-time history for each test, measured by the load cell, was recorded in LabView programs at 3000 samples per second. The engineering stress was calculated by dividing the load by the original cross-sectional area. Video of each test was captured by a high-speed camera at 3000 frames per second and later converted to files of sequential JPEGs and post-processed using proprietary software developed in-house. The engineering strain–time history of each test was produced by tracking the elongation of the central gauge length dot/line markings made on the specimens prior to testing. For each test, the failure stress (σ_f_) and corresponding strain at failure (ε_f_) were calculated. The initial Young’s modulus, E_0_, was evaluated as the slope of the first portion of the linear response. The yield stress (σ_y_) and corresponding yield strain (ε_y_) were taken at the local maximum stress after the initial linear region. The tensile toughness, U, was calculated as the total area under the stress–strain curve.

### 2.5. Experimental Testing Matrix

PVB (RA41) from Eastman and SG5000 from Kuraray were tested at temperatures of 0, 23, and 60 °C and strain rates of 2, 20, and 45 s^−1^. The experimental testing matrix is given below in Table 1.

## 3. Experimental Results

In this section, the engineering stress–strain results for the dynamic testing of PVB and SG interlayer materials at a range of strain rates and temperatures are presented. For each testing group, five valid tests were successfully completed. Subsequently, average engineering stress–strain curves were produced from the five representative samples. Average parameter values of yield stress, yield strain, failure stress, strain at failure, Young’s modulus, and tensile toughness for each testing group were calculated and are presented in Table 2.

### 3.1. PVB Interlayer

**Figure 6:**
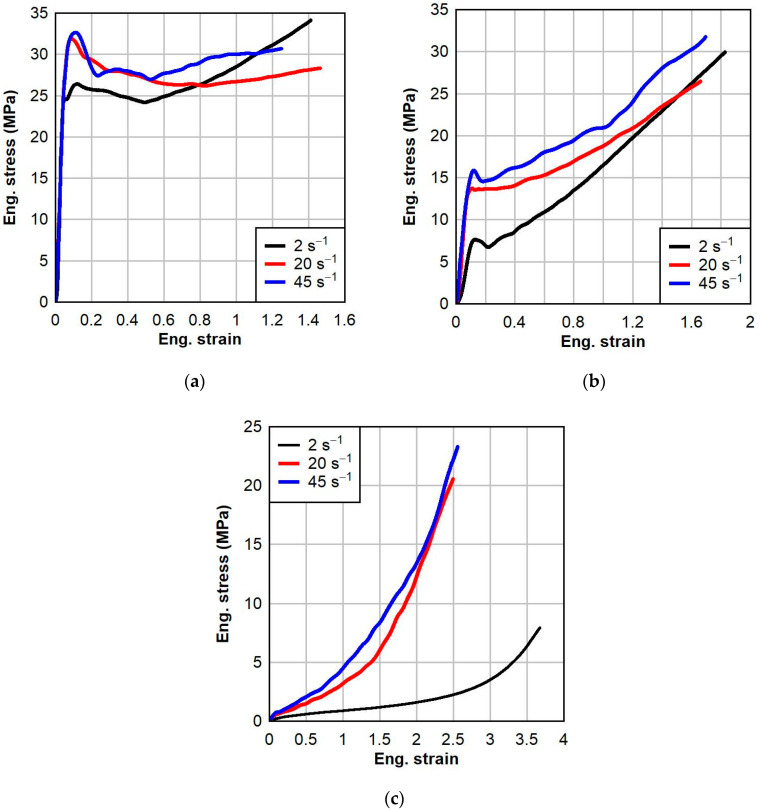
Dynamic testing results of PVB interlayer at various strain rates and temperatures: (**a**) 0 °C, (**b**) 23 °C, and (**c**) 60 °C.

The engineering stress-strain responses of PVB at three different dynamic strain rates and three test temperatures are shown in Figure 6. The effect of temperature on the nature of the behavior of PVB is apparent: at low temperature, the dynamic response of PVB is elasto-plastic; at ambient temperature, the dynamic response of PVB is viscoelastic and generally bilinear; and at elevated temperature, the dynamic response of PVB is hyperelastic. As the temperature increases, the Young’s modulus and tensile toughness decrease, and the material becomes more ductile. There is clear yielding and subsequent strain softening in the dynamic response of PVB at low and ambient temperatures, but not at elevated temperature. This is because the elevated temperature of 60 °C is greater than the glass transition temperature of PVB, which is about 50 °C. At the elevated temperature above the glass transition temperature, the polymer enters a rubbery state where the polymer chains become more mobile, allowing for greater molecular rearrangement and chain slippage.

Strain rate effects on the dynamic responses of PVB are also significant. Generally, as the strain rate increases, the yield stress, failure stress, and tensile toughness increase. These effects become more pronounced as the temperature increases. The strain rate predominantly affects the initial region of the dynamic response. As the strain rate increases, the Young’s modulus and yield stress also increase. It was found that strain rate had a negligible effect on the failure strain at low and ambient temperatures; however, the effect was significant at elevated temperatures. At elevated temperatures, the lower speed of the intermediate strain rate of 2 s^−1^ allowed sufficient time for the molecular chains to rearrange, resulting in more gradual deformation behavior and significantly greater plastic strain than at higher strain rates.

### 3.2. SG Interlayer

**Figure 7:**
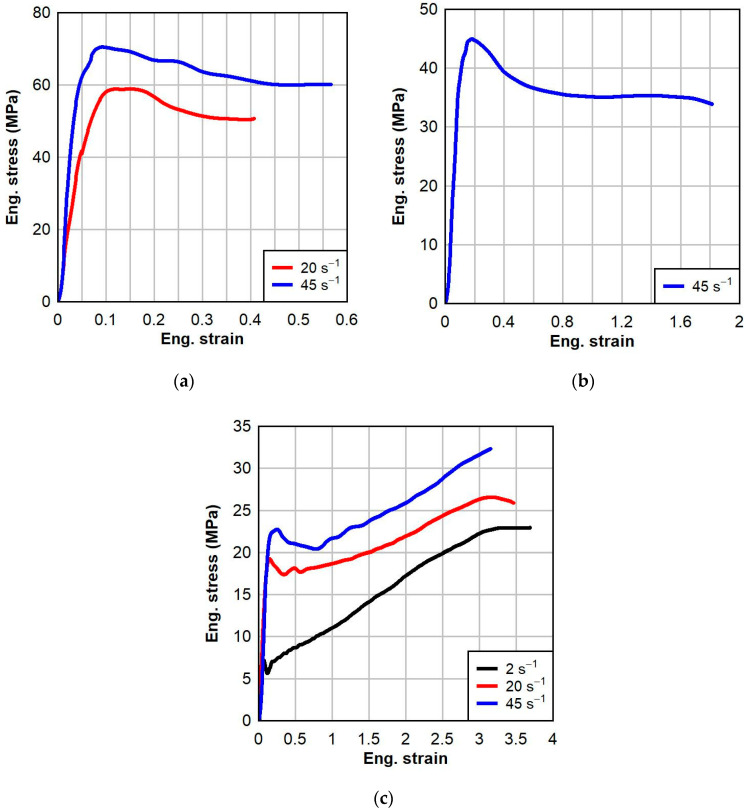
Dynamic testing results of SG interlayer at various strain rates and temperatures: (**a**) 0 °C, (**b**) 23 °C, and (**c**) 60 °C.

The engineering stress–strain responses of PVB at three different dynamic strain rates and three test temperatures are shown in Figure 7. Unlike PVB, the temperature effect did not change the nature of the dynamic response of SG. At all temperatures, the response was generally elasto-plastic, with a very stiff initial response followed by a clear yielding region and a subsequent strain-softening region. However, there was strain-hardening behavior in the large-strain response of SG at the elevated temperature. Temperature had significant impact on the failure strain of the dynamic response of SG. At low temperatures, SG became brittle and had significantly decreased failure strain. As the temperature increased, the material became much more ductile, resulting in failure strain roughly seven times greater at the elevated temperature compared to the low temperature. At the elevated temperature, the strain-softening region following yielding was much less pronounced.

Effects of the strain rate on the dynamic response of SG could be observed at the low and elevated temperatures. As the strain rate increased, the Young’s modulus and yield stress increased, resulting in greater tensile toughness. The strain rate had a greater effect on the Young’s modulus of SG at the low temperature than at the elevated temperature.

## 4. Numerical Modeling

The three-network viscoplastic (TNV) model is a general-purpose, viscoplastic constitutive model that is capable of capturing the thermo-viscoelastic response of many thermoplastics. In this section, the theoretical formulation of the TNV model is outlined. The methodology for calibrating the TNV model using PolymerFEM [54] software is detailed. Material model calibrations for the thermo-viscoelastic properties of the dynamic responses of PVB and SG interlayer materials are presented. A finite element model for simulating the dynamic tensile tests was developed and used to validate the calibrated models at different temperatures and strain rates.

### 4.1. Material Constitutive Models

**Figure 8:**
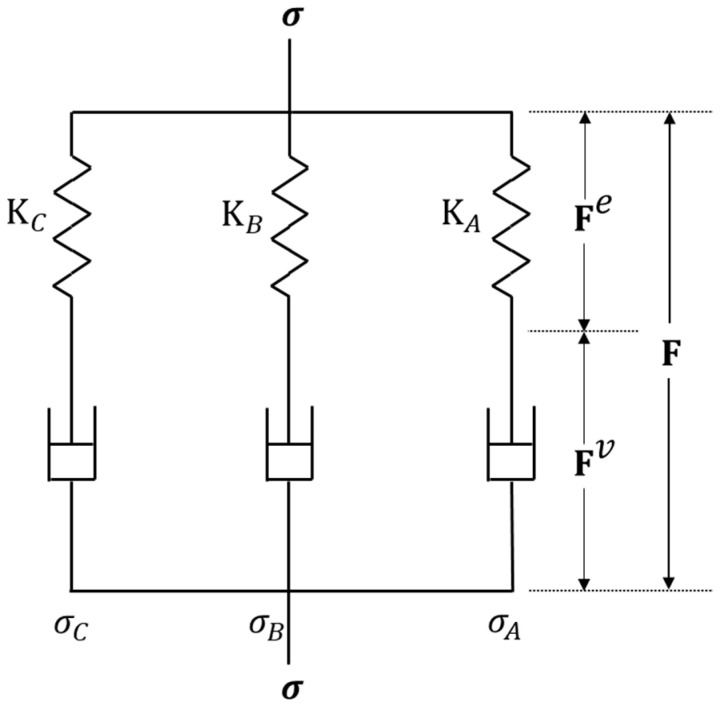
Rheological representation of the constitutive three-network viscoplastic (TNV) model.

The calibrated TNV model for the material comprised three parallel networks, as depicted in Figure 8 in rheological terms. The total deformation gradient F affected all three networks equally, F = F_A_ = F_B_ = F_C_, and the total Cauchy stress σ was the sum of stresses from each network σ = σ_A_ + σ_B_ + σ_C_. To simplify the equations in this section, we have omitted the subscripts that differentiate between the networks. This simplification is valid because networks A, B, and C all follow the same constitutive functions. The viscoplastic deformation gradient acting on each network can be separated into elastic and viscoplastic components [54].

(1) $$F=F^{e}F^{v}$$

The Cauchy stress acting on a network selected is given by the Yeoh model [53], with Cauchy stress [54]:

(2) $$\sigma =\frac{2f_{\varepsilon }\cdot \eta }{J^{e}}\left\{C_{10}+{2C}_{20}\left(I_{1}^{*}-3\right)+{3C}_{30}{\left(I_{1}^{*}-3\right)}^{2}\right\}dev\left[b^{*}\right]+\left[\kappa_{1}\left(J^{e}-1\right)+\kappa_{2}{\left(J^{e}-1\right)}^{3}+\kappa_{3}{\left(J^{e}-1\right)}^{5}\right]I$$

The Mullins damage factor $\eta_{A}$ is expressed as follows [54], where σ represents the Cauchy stress, $f_{\varepsilon }$ denotes the plastic flow evolution damage factors, [$C_{10}$, $C_{20}$, $C_{30}$] are parameters of the Yeoh model, $J^{e}$ stands for the determinant of the elastic deformation gradient, η represents the Mullins damage factor, $b^{*}$ represents the left Cauchy–Green deformation tensor, $I_{1}^{*}$ denotes the first principal invariant of the Cauchy tensor, and [$\kappa_{1}$, $\kappa_{2}$, $\kappa_{3}$] are the bulk moduli. Distortional scalars and tensors are indicated with a superscript (*).

(3) $$\sigma =1-\frac{1}{r}erf\left[\frac{U_{dev}^{\max A}-U_{dev}^{A}}{\hat{U}+\beta U_{dev}^{\max A}}\right]$$

where [r, $\hat{U}$, β] are material parameters. *η* quantifies the softening effect observed in elastomers and other crosslinked rubber-like materials during the initial loading cycles. The Mullins effect, also known as Mullins damage, occurs when an elastomer becomes slightly softer after the first few load cycles, with the mechanical response becoming more repeatable in subsequent cycles. The factor *η* is used in constitutive models to describe the extent of this softening. The variable $U_{dev}^{A}$ is the current deviatoric strain energy density in network A, and $U_{dev}^{\max A}$ is the max deviatoric strain energy density that network A has been exposed to. The Mullins damage calculation is deactivated if r = 0. The plastic flow evolution damage factor *f_εA_* is given by the deferential equation [54]:

(4) $$\frac{df_{\varepsilon A}}{dt}=\frac{1}{c_{e}}(f_{s}-f_{\varepsilon A}){\dot{\gamma }}_{A}^{v}$$

Here, [f, $c_{e}$] are material parameters, $f_{\varepsilon A}$ = 1 at *t* = 0, and ${\dot{\gamma }}_{A}^{v}$ is a constant introduced for dimensional consistency. The velocity gradient of the viscoplastic flow of network A can be written as [54]:

(5) $${\dot{F}}_{A}^{v}={\dot{\gamma }}_{0}{\left[R\left(\frac{\tau_{A}}{f_{p}\cdot f_{\varepsilon p}\cdot f_{\theta }\cdot {\hat{\tau }}_{A}}-0.001\right)\right]}^{m}F_{A}^{e-1}\left[N_{A}+bsign\left(tr\left[\sigma \right]\right)1\right]F$$

The effective stress driving the viscoplastic flow is defined by the Hill deviatoric stress [54], where [${\hat{\tau }}_{A}$, m] represent material parameters. ${\dot{\gamma }}_{0}$ = 1/s is a constant introduced for dimensional consistency. R(x)=(x+|x|)/2 denotes the ramp function; ${\hat{\tau }}_{A}$ stands for the flow resistance of the network; mmm represents the stress exponent; $f_{p}$ indicates the pressure-dependent viscoplastic flow; $N_{A}$ specifies the direction of the driving deviatoric stress; and $bsign\left(tr\left[\sigma \right]\right)1$ contributes to the volumetric viscoplastic flow, with b being a material parameter.

(6) $$\tau_{A}=\left[F\left(\sigma_{A.22}^{\prime }-\sigma_{A.33}^{\prime }\right)+G\left(\sigma_{A.33}^{\prime }-\sigma_{A.11}^{\prime }\right)+H\left(\sigma_{A.11}^{\prime }-\sigma_{A.22}^{\prime }\right)+2L\sigma_{A.23}^{\prime }+2M\sigma_{A.31}^{\prime }+2N\sigma_{A.12}^{\prime }\right]$$

If the parameter *F* = 0, then the model yields isotropically, and the effective stress is instead given by the Frobenius norm of the deviatoric stress:

(7) $$\tau_{A}=\left[\sum_{i=1}^{3}\sum_{j=1}^{3}{\left|\sigma_{A.ij}\right|}^{2}\right]$$

In these equations, $\sigma^{\prime }=dev[\sigma ]$ represents the deviatoric stress. If *F* < 1 and the other Hill parameters have their default values, then the yield stress will be lower in the 1-direction. If G < 1 and the other Hill parameters have their default values, then the yield stress will be lower in the 2-direction. And similarly, if H < 1 and the other Hill parameters have their default values, then the yield stress will be lower in the 3-direction. The tensor $N_{A}$ specifies the direction of the driving deviatoric stress of the relaxed configuration converted to the current configuration [54].

(8) $$N_{A}=\frac{dev[\sigma_{A}]}{\tau_{A}}$$

The pressure dependence of the viscoplastic flow is captured using the function [54]:

(9) $$\sigma =f\left(x\right)=\left\{\begin{array}{l} \max\left(0.5,\;1+p_{0}\frac{p}{\tau_{A}}\right),\;\;if\;p_{0}>0,\\ \max\left(0.5,\;1-p_{0}\frac{p}{\tau_{A}}\right),\;\;if\;p_{0}<0\;and\;p<0,\\ \;\;\;\;\;\;\;\;1,\;\;otherwise \end{array}\right.$$

where $p=-\frac{1}{3}tr[\sigma_{A}+\sigma_{B}+\sigma_{C}]$ is the hydrostatic pressure. In summary, if $p_{0}>0$, then the yield stress is reduced in a tensile pressure state and increased in a compressive pressure state, and if $p_{0}<0,$ then the yield stress is only reduced in a tensile pressure state. The yield evolution function $f_{\varepsilon p}$ specifies how the flow resistance evolves with the plastic strain. That dependence is captured using the following equation [54]:

(10) $$f_{\varepsilon p}=f_{f}+\left(1-f_{f}\right)exp\left[\frac{-\varepsilon_{p}}{\varepsilon_{f}}\right]$$

where $\varepsilon_{p}$ is the Mises strain obtained from ${\dot{F}}_{A}^{v}$, and [$f_{f}$, $\varepsilon_{f}$] are material parameters.

**Figure 9:**
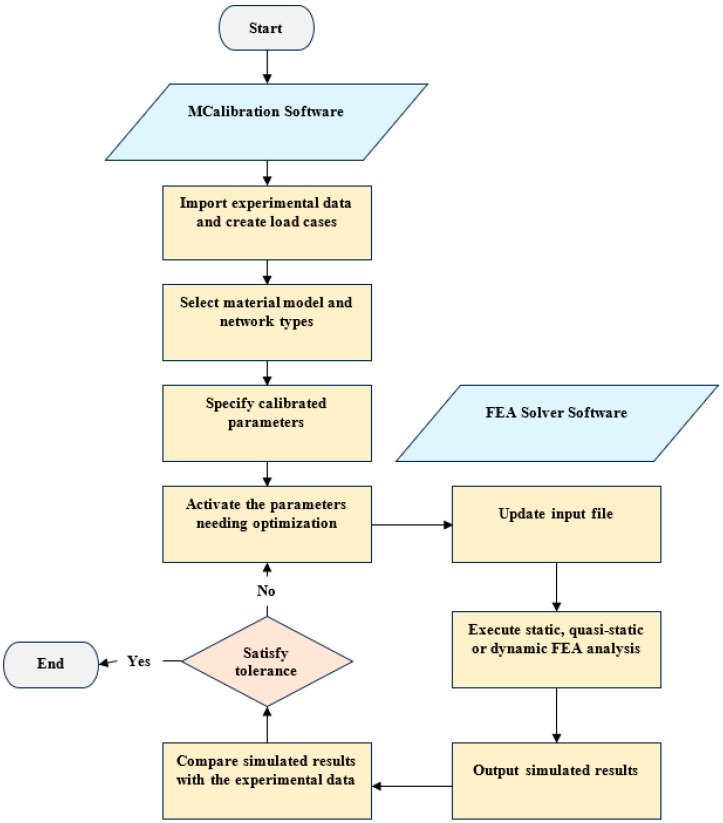
Calibration procedure flowchart.

All the TNV model parameters were calibrated based on the experimental data using the PolymerFEM software MCalibration 7.3.0. The calibration procedure flowchart, including the finite element simulations, is illustrated in Figure 9. To begin, the smoothed stress–strain responses from uniaxial tensile tests are input into MCalibration 7.3.0 as load cases. When utilizing hyperelastic models, it is critical to select the appropriate model based on the type of experimental data. Hyperelastic models may rely on the first stress invariant, *I*_1_, such as the neo-Hookean, Yeoh, and Arruda–Boyce models, or not, as in the Moony–Rivlin or Ogden models. This distinction in model classes determines the required dataset. Specifically, non- *I*_1_-based models necessitate experimental tests across multiple loading modes, while a single loading mode may suffice to calibrate *I*_1_-based models. Any previously calibrated or known material properties, such as Poisson’s ratio or Young’s modulus, can also be specified as load cases.

Next, the network types within the TNV model are selected from the PolyUMod library database depending on the type of polymer. Up to three different network types can be employed. The model supports the following hyperelastic components: Yeoh, anisotropic HGOB hyperelastic HGOB (Holzapfel–Gasser–Ogden–Bergstrom), and the hyperfoam model. Each component can be purely elastic or augmented with isotropic or anisotropic power flow and/or the Mullins damage criterion. Hyperelastic component selection is guided by material category: either thermoplastics, elastomers, thermoplastic elastomers (TPEs), or polymer foams.

**Figure 10:**
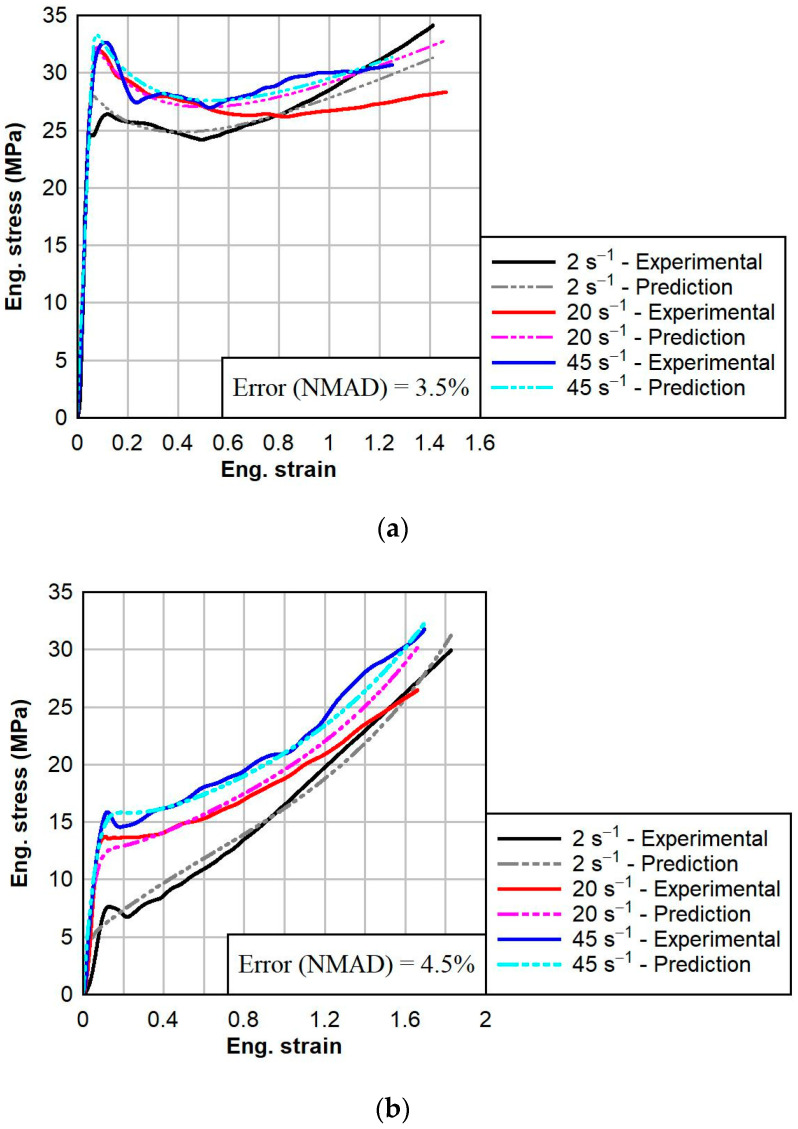

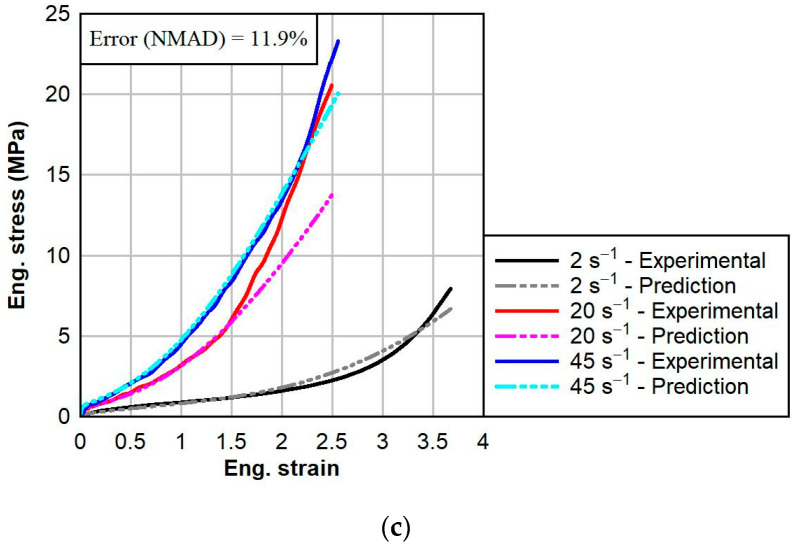
Comparison between TNV material model predictions (dotted) and tensile test experimental data (solid) for PVB at various strain rates and temperatures: (**a**) 0 °C, (**b**) 23 °C, and (**c**) 60 °C.

**Figure 11:**
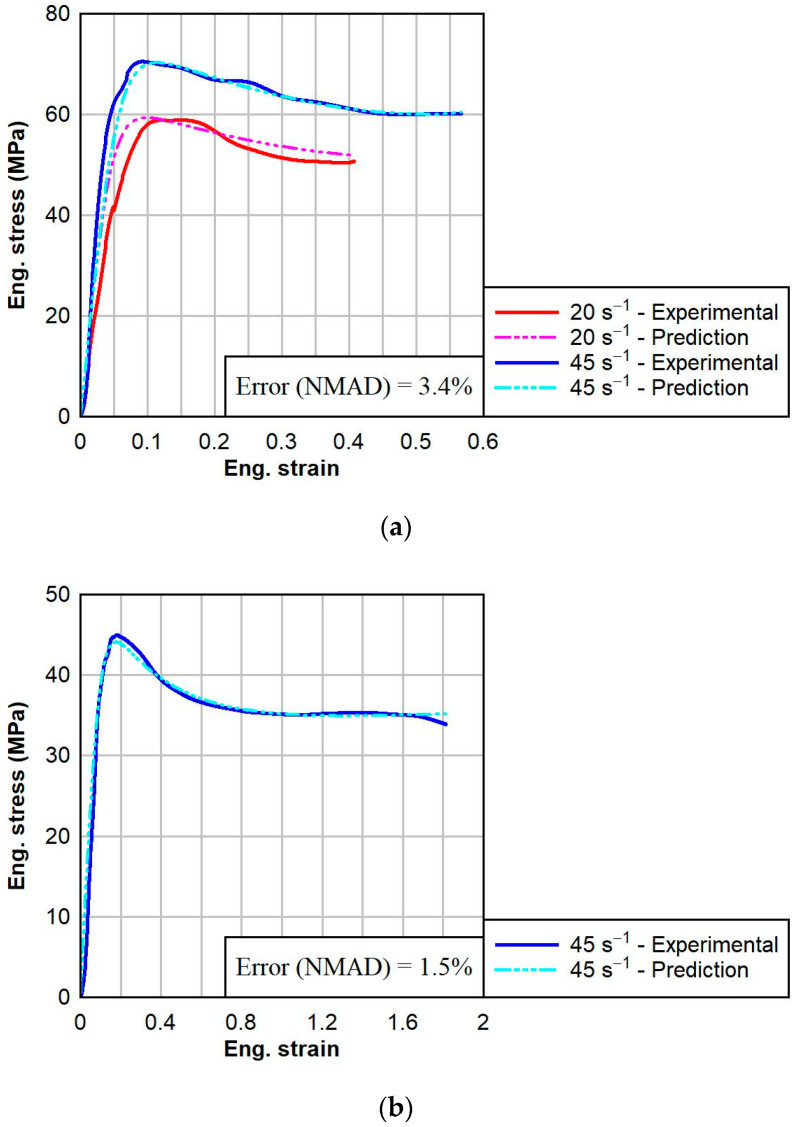

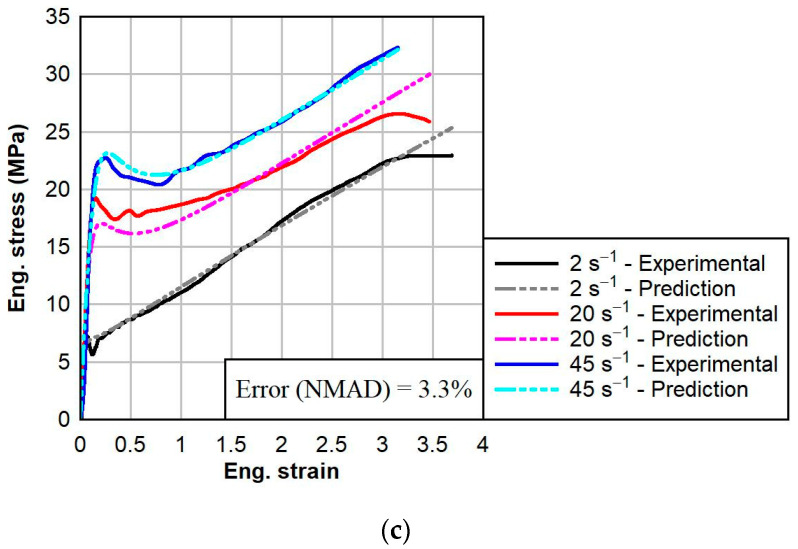
Comparison between TNV material model predictions (dotted) and tensile test experimental data (solid) for SG at various strain rates and temperatures: (**a**) 0 °C, (**b**) 23 °C, and (**c**) 60 °C.

The calibrations of the PVB and SG models were performed using a two-network configuration. The third network of the TNV model was deselected. The standard Yeoh hyperelastic model was chosen for Network 1 to capture the large strain responses of the materials. After the initial linear regions of the dynamic responses of PVB and SG, the general large strain behavior could be well defined by a simple hyperelastic model. However, viscoplastic thermoplastics present a couple of notable challenges: (1) the Young’s modulus is highly dependent on the strain rate; and (2) the large strain responses at different strain rates sometimes converge, like for the dynamic responses of PVB at ambient temperatures (Figure 10b), and at other times, the large strain responses at different strain rates do not converge, as in the case of SG at an elevated temperature (Figure 11c). Therefore, the Yeoh + Power Law Flow Cessation (PSC) model was selected for Network 2. In this model, the power exponent m is modified to be strain rate-dependent. The typical flow rule is given by the equation:

(11) $$\dot{\gamma }={\;\left(\frac{\tau }{\hat{\tau }}\right)}^{m}$$

The modified flow rule with a strain-dependent m-parameter is given by:

(12) $$\dot{\gamma }={\;\left(\frac{\tau }{\hat{\tau }}\right)}^{m^{eff}}$$

where the effective power exponent $m^{eff}$ is defined by:

(13) meff=mi−mf−ϵpϵ^+mf

In this formulation, the power exponent $m^{eff}$ is controlled by two parameters, $m_{i}$ and $m_{f}$, which can be optimized to fit the level of strain rate dependence of the Young’s modulus. The flow rate is further optimized to control the large strain behavior by inclusion of the flow cessation factor $f_{v}$. In this approach, the flow rate is given by the following equation:

(14) $${\dot{\gamma }}^{p}={\dot{\gamma }}_{0}\cdot f_{v}\cdot {\;\left(\frac{\tau }{\hat{\tau }}\right)}^{m^{eff}}$$

where ${\dot{\gamma }}_{0}\;$= 1/s is a constant introduced for dimensional consistency, and the flow cessation factor $f_{v}$ is given by:

(15) $$f_{v}=1-A\cdot \left[1-\frac{\beta -B}{1-B}\cdot {\left(\beta \right)}^{m^{eff}}\right]$$

The β parameter specifies the normalized molecular orientation angle. Note that, with this definition, if A = 0, then the flow cessation is deactivated, and if *A* = 1, then the flow cessation becomes the same as the equation proposed by Dupaix and Boyce. In other words, A can be used to scale how strong the flow cessation should be.

After the selection of network types, a couple of intrinsic parameters needed to be input. The mass densities of PVB and SG were set to 0.95 and 1.07 g/cm^3^, respectively [24]. The Poisson’s ratios for PVB and SG were set as load cases to 0.495 and 0.45, respectively [25,26].

Optimization can be carried out using several different numerical methods, such as a random search, a genetic search, a global optimum search, etc. After selection of the material model and network types, initial parameter predictions are generated using the MCalibration 7.3.0 software. The parameters which need optimization are selected by the user, and lower and upper bounds can be modified or deselected for each parameter. Once the initial parameters were selected and bounds were set, an automatic extensive optimization was performed, which incorporated an initial random search, the Levenberg–Marquardt search, and the new unconstrained optimization with quadratic approximation (NEWUOA) algorithm. The optimizations performed in this study targeted the normalized mean absolute difference, although it is important to note that several statistical metrices, including the mean square difference or the coefficient of determination, can be utilized. For SG, a fitness weight function of $f\left(x\right)=5\exp\left(-x\right)+1$ was utilized to prioritize the fit of the initial response and softening region. The calibrated models were then exported into Ansys LS-DYNA [62] format and applied to the geometry as user-defined material models.

The calibrations for PVB and SG at each temperature are shown below in Figure 10 and Figure 11, respectively. For each calibration, the normalized mean absolute difference (NMAD) fitness function was chosen. The calibrations resulted in good fits to the experimental data, with the average NMAD errors for PVB and SG being 6.6% and 3.1%, respectively. The calibrated model parameters for PVB at 0 °C, 23 °C, and 60 °C are given below in Table 3. The calibrated model parameters for SG at 0 °C, 23 °C, and 60 °C are given below in Table 4.

### 4.2. Finite Element Model Components

**Figure 12:**
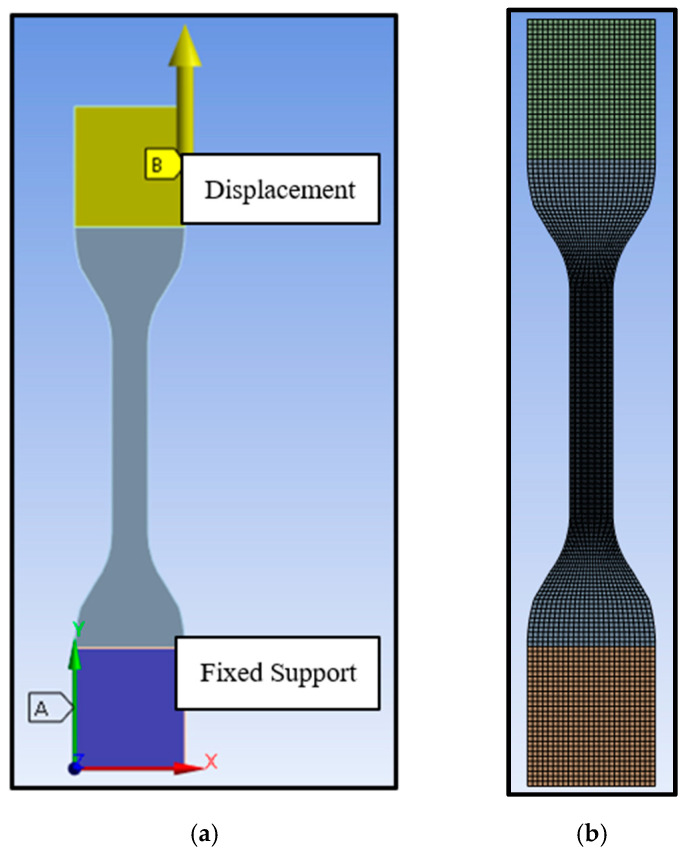
Ansys LS-DYNA model: (**a**) FE model boundary conditions and (**b**) specimen meshing.

A finite element computational model was built in the Ansys LS-DYNA to simulate the tensile test under displacement control conditions. The tensile test specimen geometry and boundary conditions are shown in Figure 12a. All nodes of the top grip shown in yellow were restrained in the transverse directions and permitted to move only in the longitudinal direction. All nodes in the bottom grip shown in blue were fixed in all directions. The displacement was applied to the top grip, which caused the specimen to deform in the vertical direction only. Hexahedral eight-node linear elements (HEX8) with 0.635 mm mesh sizes were used (Figure 12b). A convergence study was conducted, and the model converged at a 0.635 mm element size. The PolymerFEM software was integrated into Ansys LS-DYNA to define the material models and input all calibrated material model parameters.

### 4.3. Material Model Validations and Discussion

In order to validate that the calibrated three-network viscoplastic (TNV) models were capable of simulating the thermo-viscoelastic responses of PVB and SG, finite element simulations of tensile tests at the experimental test temperatures and strain rates were performed. The simulations were performed using displacement control and isothermal temperature fields. To accurately replicate the experimental tests, two nodal displacement probes were used to measure the longitudinal displacement of the central 25.4-mm (1-inch) gage length, and the engineering strain history was derived from these nodal displacements. The dynamic force reaction was measured at the bottom fixed support, which is analogous to the load cell used in experimental testing, and the engineering stress history was calculated as the force reaction divided by the original cross-section of the central gage length. For each temperature, simulations were performed at strain rates of 2 and 45 s^−1^.

**Figure 13:**
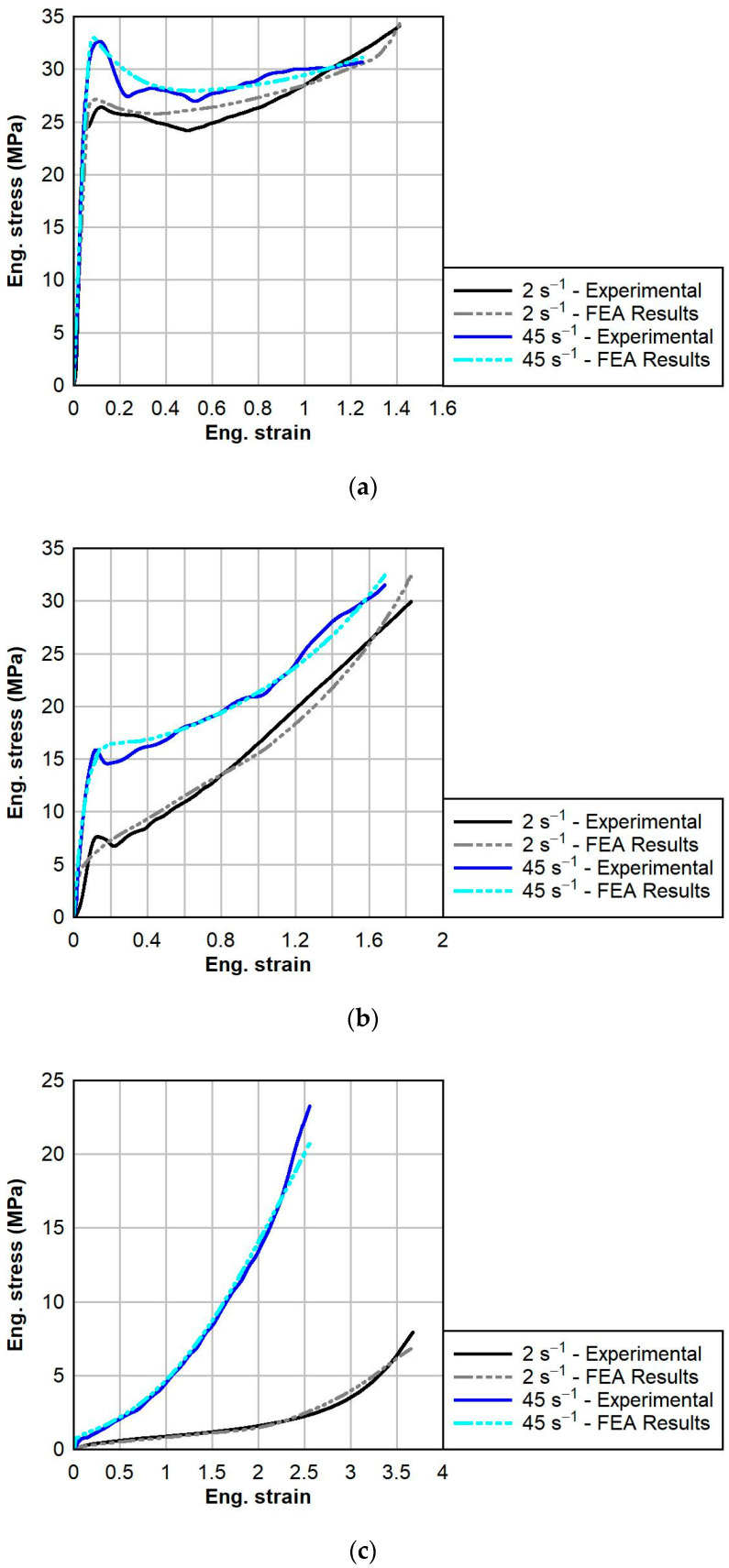
Comparison between TNV model FEA results (dotted) and tensile test experimental data (solid) for PVB at various strain rates and temperatures: (**a**) 0 °C, (**b**) 23 °C, and (**c**) 60 °C.

**Figure 14:**
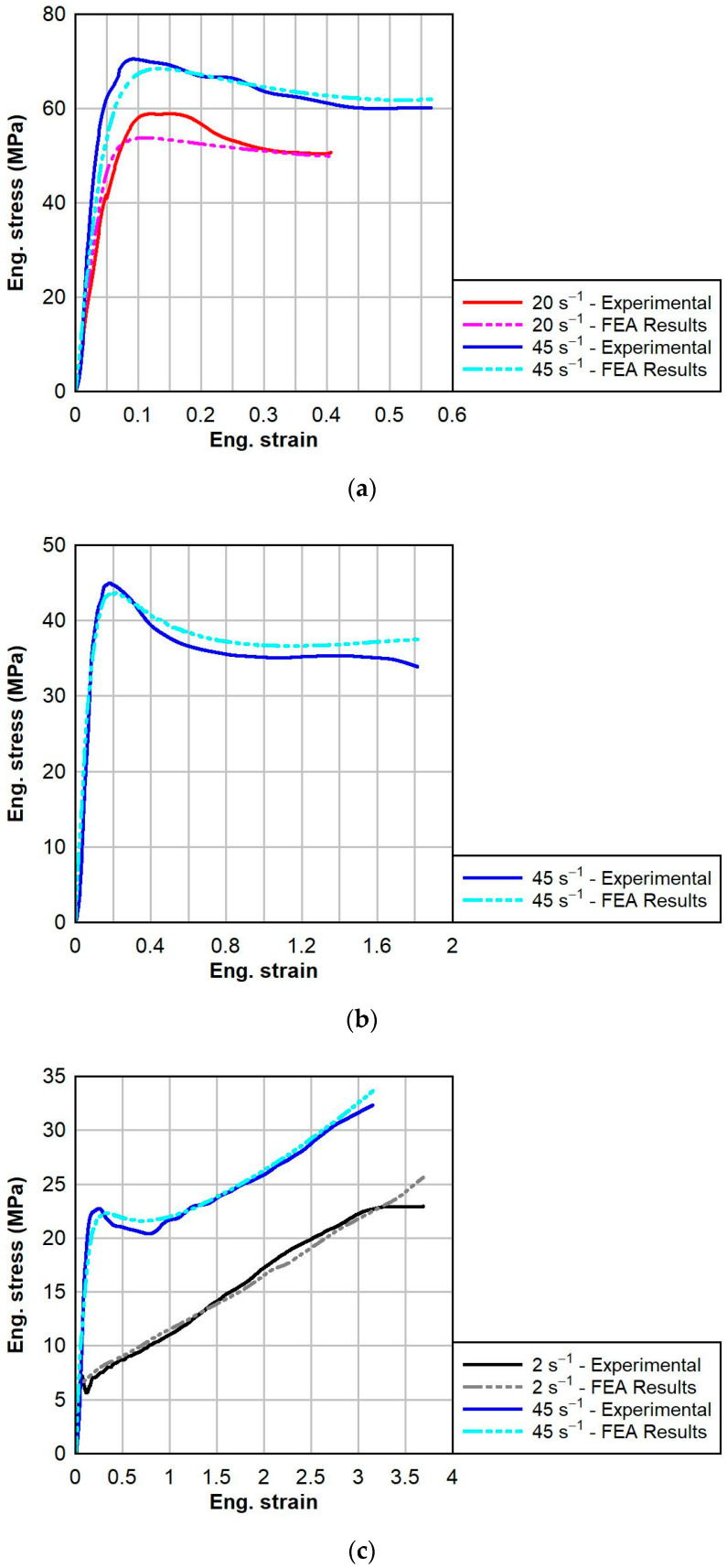
Comparison between TNV model FEA results (dotted) and tensile test experimental data (solid) for SG at various strain rates and temperatures: (**a**) 0 °C, (**b**) 23 °C, and (**c**) 60 °C.

The comparisons between the finite element analysis results and the experimental testing results for PVB and SG are shown below in Figure 13 and Figure 14, respectively. The models predictions agreed well overall with the experimental data and were able to capture substantial changes in behavior due to temperature and strain rate. The fitness of each prediction was characterized by the normalized mean average difference (NMAD) percentage between the finite element analysis test results and experimental data, given in Table 5. The NMAD for each test group was less than 10%. The NMAD was highest for the PVB at 60 °C group, which was similar to the calibration, due to the extreme strain rate effect on the response of PVB at temperature greater than the glass transition temperature. The material model for SG accurately captured the post-peak softening behavior at 0 and 23 °C and the subsequent hardening response of SG at 60 °C. Overall, the constitutive model predictions demonstrate the suitability of the calibrated material models to simulate the thermo-viscoelastic dynamic responses of PVB and SG.

## 5. Conclusions and Future Perspectives

In this study, the thermo-viscoelastic dynamic behaviors of PVB- and SG-laminated glass interlayer materials under monotonic loading conditions were investigated experimentally. A drop weight testing machine was modified to perform dynamic testing of the polymers at high strain rates of 20 and 45 s^−1^. A pneumatic testing machine was fabricated to perform intermediate strain rate testing at 2 s^−1^. Both testing machines were outfitted with a temperature enclosure to perform isothermal testing at temperatures of 0, 23, and 60 °C. The experimental engineering stress–strain data were used to calibrate temperature- and strain rate-dependent material models for PVB and SG using the three-network viscoplastic (TNV) constitutive model. A representative finite element model of the tensile tests performed was constructed, and simulations were performed at each temperature and multiple strain rates to validate the calibrated material models. The main findings of this study are as follows:

- Strain rate predominantly affected the initial responses of PVB and SG. Initial modulus and yield stress increased with strain rate, resulting in increased toughness.
- Temperature affected the characteristic nature of the response of PVB, but not for SG. As temperature increased, the response of PVB transitioned from elastoplastic to bilinear to hyperelastic. At all temperatures, SG interlayer specimens exhibited a linear response followed by yield; strain softening; plastic flow; and, finally, strain hardening at large strains, achieved at elevated temperatures.
- In general, PVB and SG became stiffer and less ductile as the temperature decreases. The temperature effects were more pronounced in SG than in PVB. For a strain rate of 45 s^−1^, as the temperature decreased from 23 °C to 0 °C, the failure strain of SG decreased by 69% and the initial modulus increased by 418%, whereas for PVB, the failure strain decreased by 26% and the initial modulus increased by 200%.
- There was clear yielding and subsequent strain softening in the dynamic response of PVB at 0 °C and 23 °C, but not at 60 °C. The elevated temperature of 60 °C was greater than the glass transition temperature of PVB of about 50 °C, and therefore, the behavior was dominated by the viscous component of the viscoelastic response.
- Temperature- and strain rate-dependent material models for the dynamic responses of PVB and SG interlayer materials were calibrated using the three-network viscoplastic (TNV) model, with average normalized mean absolute differences (NMADs) between the experimental data and model predictions of 6.6% and 2.7% for PVB and SG, respectively.
- The FEA results based on the calibrated material models were successfully able to predict the thermal viscoelastic dynamic behaviors of PVB and SG interlayer materials under tensile loading, with average NMADs between the FEA results and experimental data of 6.0% and 4.9% for PVB and SG, respectively.

In the future, additional investigations are needed to improve the material models for their particular use of modeling laminated glass systems. The calibrations at each temperature can be used to develop combined multi-temperature material models to predict the response of the material at any temperature or strain rate within the bounds of the experimental data. Additionally, future work will focus on incorporating an analytical approach similar to the WLF model to account for the temperature dependence of material properties. This enhancement aims to provide a more comprehensive understanding and accurate prediction of the material behavior under varying thermal conditions. During the initial response of laminated glass before glass cracking, the interlayer material acts to transfer shear force between the glass panels during bending. Therefore, the addition of simple shear test data at multiple temperatures to the calibrations can improve the ability of the material models to predict the initial bending response of laminated glass.

## Figures and Tables

**Table 1:** Experimental testing matrix.

Material/Manufacturer	Temperature (°C)	Strain Rates (s^−1^)
PVB (RA41)/Eastman (Eastman Chemical Company, Kingsport, TN, USA)	0	2, 20, 45
	23	2, 20, 45
	60	2, 20, 45
SG5000/Kuraray (kuraray, Houstan, TX, USA)	0	20, 45
	23	45
	60	2, 20, 45

**Table 2:** Polymer testing results.

Polymer	Temperature (°C)	Strain Rate (s^−1^)	Yield Stress (MPa)	Yield Strain (mm/mm)	Failure Stress (MPa)	Failure Strain (mm/mm)	Young’s Modulus (MPa)	Toughness (MPa-mm/mm)
PVB	0	2 s^−1^	26.4	0.122	34.1	1.412	519	37.9
		20 s^−1^	31.9	0.088	28.3	1.464	573	39.6
		45 s^−1^	32.7	0.111	30.7	1.249	586	35.5
	23	2 s^−1^	7.6	0.129	30.0	1.828	63.2	29.2
		20 s^−1^	13.8	0.108	26.5	1.661	194	29.6
		45 s^−1^	15.9	0.120	31.8	1.694	195	35.1
	60	2 s^−1^	-	-	7.9	3.677	2.3	7.8
		20 s^−1^	-	-	20.6	2.496	7.0	16.4
		45 s^−1^	-	-	23.3	2.558	8.7	20.8
SG5000	0	20 s^−1^	58.8	0.120	50.7	0.407	1035	20.3
		45 s^−1^	70.5	0.092	60.2	0.567	2203	34.9
	23	45 s^−1^	45.0	0.128	33.9	1.814	425	64.9
	60	2 s^−1^	7.3	0.067	22.9	3.690	165	58.1
		20 s^−1^	19.3	0.147	25.9	3.468	191	73.9
		45 s^−1^	22.7	0.249	32.3	3.154	242	77.2

**Table 3:** Calibrated material model parameters for PVB at 0 °C, 23 °C, and 60 °C.

			0 °C		23 °C		60 °C	
Description	Symbol	Units	Network A	Network B	Network A	Network B	Network A	Network B
Yeoh parameter 1	$C_{10}$	MPa	5.24	90.1	3.39	39.3	0.169	132
Yeoh parameter 2	$C_{20}$	MPa	0	0	0	0	0	0
Yeoh parameter 3	$C_{30}$	MPa	1.92 × 10^−5^	0	0.021	0	1.32 × 10^−7^	0
Bulk modulus 1	$\kappa_{1}$	MPa	922	922	242	242	3.87	3.87
Bulk moduli 2 and 3	$\kappa_{2},\;\kappa_{3}$	MPa	0	0	0	0	0	0
Flow resistance of network	$\hat{\tau }$	MPa	0	20.7	0	1.69	0	0.035
Initial stress exponent	$m_{i}$	-	0	15.7	0	2.40	0	1.72
Final stress exponent	$m_{f}$		0	20	0	5.31	0	0.064
Transition strain power exponent	$\varepsilon_{m}$		0	0.548	0	1.19	0	0.85
Volumetric flow coefficient	b	-	0	0	0	0	0	0
Pressure dependence of flow	$p_{0}$	-	0	0	0	0	0	0
Yield evolution	$f_{f}$	-	0	0.100	0	1.48	0	1.64
Yield evolution strain	$\varepsilon_{f}$	-	0	0.550	0	0.010	0	0.013
Flow damage strain	$c_{\varepsilon }$	-	0	0.1	0	0.1	0	0.1
Flow damage final state	$f_{s}$	-	0	1	0	1	0	1
Strength of flow cessation	A		0	1	0	1	0	0.994
Normalized cutoff orientation for flow cessation	B		0	0	0	0	0	0.180

**Table 4:** Calibrated material model parameters for SG at 0 °C, 23 °C, and 60 °C.

			0 °C		23 °C		60 °C	
Description	Symbol	Units	Network A	Network B	Network A	Network B	Network A	Network B
Yeoh parameter 1	$C_{10}$	MPa	2.44	229	1.82	84.2	2.44	32.5
Yeoh parameter 2	$C_{20}$	MPa	0	0	0	0	0	0
Yeoh parameter 3	$C_{30}$	MPa	1.35	0	0.0009	0	5.02 × 10^−8^	0
Bulk modulus 1	$\kappa_{1}$	MPa	2050	2050	837	837	338	338
Bulk moduli 2 and 3	$\kappa_{2},\;\kappa_{3}$	MPa	0	0	0	0	0	0
Flow resistance of network	$\hat{\tau }$	MPa	0	15.4	0	11.9	0	3.47
Initial stress exponent	$m_{i}$	-	0	2.89	0	3.25	0	3.97
Final stress exponent	$m_{f}$		0	4.41	0	3.25	0	2.22
Transition strain power exponent	$\varepsilon_{m}$		0	2.93	0	0.039	0	1.27 × 10^−4^
Volumetric flow coefficient	b	-	0	0	0	0	0	0
Pressure dependence of flow	$p_{0}$	-	0	0	0	0	0	0
Yield evolution	$f_{f}$	-	0	0.955	0	0.257	0	0.341
Yield evolution strain	$\varepsilon_{f}$	-	0	0.431	0	0.885	0	0.498
Flow damage strain	$c_{\varepsilon }$	-	0	0.1	0	0.1	0	0.1
Flow damage final state	$f_{s}$	-	0	1	0	1	0	1
Strength of flow cessation	A		0	0.730	0	0.996	0	1
Normalized cutoff orientation for flow cessation	B		0	0.054	0	0	0	0

**Table 5:** Fitness of the calibrated TNV material models for PVB and SG5000 given by normalized mean absolute difference (NMAD) (%) for each testing group.

Polymer	Temperature (°C)	Strain Rate (s^−1^)	Normalized Mean Absolute Difference, NMAD (%)
PVB	0	2 s^−1^	4.37
		45 s^−1^	2.28
	23	2 s^−1^	7.72
		45 s^−1^	4.28
	60	2 s^−1^	9.80
		45 s^−1^	7.23
SG5000	0	20 s^−1^	6.47
		45 s^−1^	4.40
	23	45 s^−1^	5.88
	60	2 s^−1^	4.33
		45 s^−1^	3.56

## Data Availability

Data are contained within the article.
